# Mutated Hif-1αa Proteins with Increased Stability Under Normoxic Conditions Enhance Hypoxia Tolerance of Otomorphs by Promoting Glycolysis and Lactate Shuttle

**DOI:** 10.3390/ani16010119

**Published:** 2025-12-31

**Authors:** Xianzong Wang, Junli Yan, Huili Zhai, Jiali Guo, Xueyi Wang, Qing Liu, Shaozhen Liu

**Affiliations:** 1 College of Animal Science, Shanxi Agricultural University, Taigu District, Jinzhong 030801, China; zhaihuili_l@163.com (H.Z.); 17302250363@163.com (J.G.); 15685488103@163.com (X.W.); liuqing_sxau@126.com (Q.L.); 2 Shanxi Key Laboratory of Animal Genetics Resource Utilization and Breeding, Shanxi Agricultural University, Taigu District, Jinzhong 030801, China; 3 College of Urban and Rural Construction, Shanxi Agricultural University, Taigu District, Jinzhong 030801, China; y_jl2020@163.com

**Keywords:** hypoxia-inducible factor, whole-genome duplication, LXXLAP motif, dissolved oxygen, expression, mitochondrion, oxygen-labile α subunit

## Abstract

Fishes are frequently exposed to hypoxic stress, yet their tolerance to hypoxia varies significantly among species. The association between this variation and alterations in the hypoxia-inducible factor (HIF) pathway remains unclear. We discovered that otomorphs generally retain two Hif-1α paralogs (Hif-1αa and Hif-1αb), resulting from the teleost-specific genome duplication (TGD), whereas most euteleosts possess only a single Hif-1αa copy. In otomorphs, key mutations disrupt one conserved Leu-X-X-Leu-Ala-Pro (LXXLAP) motif in the oxygen-dependent degradation (ODD) domain of the Hif-1αa proteins. Molecular dynamics simulations revealed that these mutations impede the recognition of the critical proline residue by prolyl hydroxylase domain protein 2 (PHD2), suggesting enhanced normoxic stability of Hif-1αa. We also investigated the expression profiles of *hif-1α* and downstream genes in four fish species (two otomorphs and two euteleosts). In otomorphs, the *hif-1αa* genes were highly expressed specifically in the heart; concomitantly, two critical downstream genes, *ldha* and *mct4*, exhibited relatively high expression levels in vital tissues such as the heart, brain, and muscle. This coordinated expression pattern promotes a heightened glycolytic capacity and facilitates lactate shuttling in these tissues, thereby ensuring energy supply during hypoxic stress. Our integrated computational analyses indicate that otomorphs achieve enhanced hypoxia tolerance through the subfunctionalization of Hif-1α paralogs.

## Simple Summary

Otomorpha fishes generally possess two copies of Hif-1α (Hif-1αa and Hif-1αb), resulting from the teleost-specific genome duplication. Mutations in the LXXLAP motif of Hif-1αa confer increased stability under normoxic conditions. This enhanced stability enables vital tissues, including the heart, brain, and muscle, to sustain high glycolytic rates, thereby ensuring an adequate energy supply during hypoxic stress. These findings are derived from integrated computational analyses including comparative genomics, molecular dynamics simulations, and transcriptome data mining.

## 1. Introduction

The concentration of dissolved oxygen (DO) in aquatic environments is substantially lower and more prone to fluctuation than in the terrestrial atmosphere [1,2]. Consequently, fishes frequently encounter hypoxic conditions. In aquaculture, water DO levels must be closely monitored to prevent mass mortality due to hypoxia. However, hypoxia tolerance varies dramatically among fish species. For instance, goldfish (*Carassius auratus*) can maintain activity under anaerobic conditions until glycogen reserves are depleted and thrive at relatively low DO concentrations [3,4,5]. In contrast, species such as rainbow trout (*Oncorhynchus mykiss*) require near-saturated DO for optimal growth [6,7]. Therefore, comparing the molecular mechanisms between hypoxia-tolerant and -intolerant fishes is highly valuable, as it may reveal strategies for enhancing hypoxia tolerance in susceptible species.

The hypoxia-inducible factor (HIF) pathway is a central mechanism enabling animals to sense and respond to cellular hypoxia [8,9]. Its core components—HIF-1α, HIF-1β (also known as aryl hydrocarbon receptor nuclear translocator, ARNT), prolyl hydroxylase domain protein 2 (PHD2), and von Hippel-Lindau (VHL)—are conserved from nematodes to mammals [8,10]. ARNT is stable and constitutively expressed, whereas the stability of the HIF-1α subunit is tightly regulated by the PHD-VHL system [11]. Unlike ARNT, the HIF-1α subunit contains an additional oxygen-dependent degradation (ODD) domain, which typically includes two conserved Leu-X-X-Leu-Ala-Pro (LXXLAP) motifs (one N-terminal and one C-terminal) [10,12]. Under normoxic conditions, the critical proline residue within these motifs is hydroxylated by PHD2. This modification enables HIF-1α to be recognized by the VHL E3 ubiquitin ligase complex, leading to its polyubiquitination and rapid proteasomal degradation [9]. Consequently, under normoxia, HIF-1α has an extremely short half-life, estimated at only 5–10 min [13]. Under hypoxic conditions, diminished PHD2 activity allows HIF-1α to accumulate and dimerize with ARNT in the nucleus. The active HIF-1 complex binds to hypoxia response elements (HREs) in the regulatory regions of target genes, inducing the transcription of hundreds of genes involved in the hypoxic response [14,15]. These downstream genes promote processes such as enhanced glycolysis, angiogenesis, and erythropoiesis, thereby ensuring energy supply and improving the efficiency of oxygen and fuel delivery during hypoxia [16].

Given the central role of the HIF pathway in hypoxic response, interspecific variation in its regulation, potentially arising from evolutionary events such as gene duplication, could underpin differences in hypoxia tolerance among fishes. Although the HIF pathway is highly conserved, its complexity has increased during animal evolution [8,10]. For example, vertebrates acquired paralogs of HIF-1α and PHD2 (i.e., HIF-2α, HIF-3α, PHD1, and PHD3) through whole-genome duplication (WGD) events in their common ancestor [17,18]. These additional copies can form more diverse HIF complexes, enabling finer regulation of downstream gene expression. The common ancestor of teleosts underwent a teleost-specific genome duplication (TGD) event [19]. Subsequent lineage-specific WGDs occurred in the common ancestors of goldfish and common carp (*Cyprinus carpio*), and independently in salmonids [20,21]. One study reported that all examined Otomorpha species retain two *hif-1α* copies [22]. Such retained paralogs often undergo subfunctionalization or neofunctionalization, adding complexity to the regulatory network [23,24]. Notably, species such as zebrafish (*Danio rerio*), goldfish, and many catfish—all belonging to the Otomorpha cohort [25]—exhibit remarkable hypoxia tolerance. This observation leads us to hypothesize that the widespread hypoxia tolerance in otomorphs might be tightly correlated with their retention of two *hif-1α* copies.

In this study, we first compared *hif-1α* gene copy number variation across major fish lineages. We then identified key mutations in the otomorphs Hif-1αa proteins and investigated their impact on protein stability using molecular dynamics simulations. Furthermore, we correlated the expression profiles of *hif-1α* with those of key downstream HIF target genes across different species. Finally, by integrating results from these computational analyses, we propose a mechanism for hypoxia pre-adaptation in Otomorpha fishes.

## 2. Materials and Methods

### 2.1. Identification of Homologs

The human HIF-1α sequence (GenBank accession No. NP_001230013.1) was used as the query to identify homologs in fish species. Non-redundant protein sequences for mouse (*Mus musculus*), chicken (*Gallus gallus*), and 33 fish species were downloaded from the NCBI FTP site (https://ftp.ncbi.nlm.nih.gov/genomes/refseq/, accessed on 26 September 2025; files with the suffix “protein.faa.gz”). The correspondence between protein accession numbers and gene IDs was obtained from the “gene2accession” file (downloaded from https://ftp.ncbi.nlm.nih.gov/gene/DATA/, accessed on 26 September 2025). For genes with multiple splice variants, the longest protein isoform was selected for analysis. These sequences were used to construct a local BLAST database (ncbi-blast-2.15.0+). A BLASTP search was performed against this database using the human HIF-1α query, with the maximum target sequences set to 1000 and an e-value threshold of 1 × 10^−5^. Hits with a pairwise query-hit sequence coverage exceeding 30% were retained. The protein sequences of the query and retained hits were combined and aligned with MAFFT (v7.427) [26], using the L-INS-i method. A phylogenetic tree was then inferred from the alignment with RAxML (v8.2.8) [27] under the GAMMA model of rate heterogeneity, using an automatically selected substitution model and 500 bootstrap replicates.

For all genes of interest, their chromosomal neighbors were identified by sorting the starting positions of genes on the same chromosome (using data from the “gene2accession” file). Based on gene tree topology and synteny (the distribution of gene neighbors), sequences that were not orthologs of the seed sequence were manually identified and discarded using ETE3 (v3.1.1) [28]. The remaining sequences were used for a second multiple sequence alignment, from which the final phylogenetic tree was inferred.

The same procedure was used to identify homologs of PHD2 and known downstream genes of HIF-1α in zebrafish, goldfish, medaka, rainbow trout, mouse, and chicken.

### 2.2. Identification of Conserved Domains

A batch conserved domain search (CD-search; https://www.ncbi.nlm.nih.gov/Structure/bwrpsb/bwrpsb.cgi, accessed on 1 October 2025) was conducted to identify conserved domains in all selected protein sequences, using the default parameters. For a given protein sequence, when overlaps between predicted conserved domains were encountered, the hit with the higher (less significant) e-value was discarded. For visualization alongside the phylogenetic tree, the start and end positions of conserved domains were mapped to their corresponding positions in the multiple sequence alignment. The locations of LXXLAP motifs were determined based on a previous study [29] and the multiple sequence alignment.

### 2.3. Protein Complex Modeling

Structures of PHD2.HIF-1α complexes (Supplementary Table S1) were predicted using AlphaFold (v2.3.1) [30,31]. Template complex of PHD2.HIF-1α, containing 2-oxoglutarate (2OG) and Mn(II), was obtained from a crystal structure (PDB ID: 5L9B). Each predicted complex was aligned to the template complex based on the PHD2 part. The HIF-1α peptide in the predicted complex was then trimmed, and all amino acid residues in the template were deleted using PyMOL (v2.5.0) [32]. This procedure allowed for the transfer of the 2OG and Mn(II) coordinates from the template to the predicted complexes. As zebrafish Hif-1αa lacks a conserved LXXLAP motif in the N-terminal of the ODD domain (NODD), we constructed a comparative Phd2.Hif-1αa_NODD_ by mutating corresponding residues in Phd2.Hif-1αb_NODD_. This ensured that the single proline residue in the mutated LXXLAP motif was positioned near the 2OG and Mn(II) in the active site.

An ACE cap was added to the N-terminus and an NME cap to the C-terminus of each peptide. The Mn(II) ion was replaced with Fe(II). Hydrogen atoms were added to the 2OG ligand at pH 7.0 using Avogadro (v1.2.0) [33]. Parameters for 2OG were then generated using CGenFF (v2.5) [34]. All penalty scores were below 10, confirming that the derived topology and parameters for 2OG were reliable.

### 2.4. Molecular Dynamics Simulations

Molecular dynamics (MD) simulations were performed using GROMACS (2023.1) [35], largely following established protocols [36]. The CHARMM36 all-atom force field and the TIP3P water model were used [37]. Each complex was solvated in a periodic rhombic dodecahedral box, with a minimum distance of 12 Å between the solute and the box edge. Ions were added to neutralize the system’s net charge and to achieve a physiological ion concentration of 0.15 M NaCl. All systems were energy minimized using the steepest descents method until the magnitude of the potential energy gradient was 1000.0 kJ mol^−1^ nm^−1^ or smaller. The systems were subsequently equilibrated in two phases: 1000 ps under an NVT ensemble (constant Number of particles, Volume, and Temperature) to stabilize the temperature at 300 K, followed by 1000 ps under an NPT ensemble (constant Number of particles, Pressure, and Temperature) to stabilize the pressure at 1 bar. Production MD simulations were then run for 400 ns each, with coordinates saved every 10 ps. For each system, six independent replicas were performed, starting from the energy minimization step. Interatomic distances were calculated using built-in GROMACS tools.

### 2.5. Gene Expression Analyses

To obtain tissue expression profiles of genes in zebrafish, goldfish, medaka (*Oryzias latipes*), and rainbow trout, we screened relevant BioProjects for each species. We selected projects with high-quality data that included sampling from multiple tissues (Supplementary Table S2). Links to download raw RNA-seq data were obtained by searching the SRA Run Selector (https://www.ncbi.nlm.nih.gov/Traces/study/, accessed on 22 October 2025) using BioProject accession numbers as queries. Compressed raw reads were converted to FASTQ format using fasterq-dump from the SRA Toolkit (v3.0.5). These reads were then processed with fastp (v0.23.4) [38] using default parameters to remove low-quality reads, adapters, and other contaminants. Reference transcriptome and genome sequences for the four species were downloaded from the NCBI RefSeq database (https://ftp.ncbi.nlm.nih.gov/genomes/refseq/, accessed on 22 October 2025; files with suffixes “rna.fna.gz” or “genomic.fna.gz”). These sequences were used to build SAF-formatted genome indices for each species using salmon index [39]. Transcript abundances were quantified for each tissue sample using salmon quant. The correspondence between mRNA accession numbers and NCBI gene IDs was obtained from the “gene2accession” file. For each gene, the transcripts per million (TPM) values for all splice variants were summed to obtain the overall gene-level TPM.

## 3. Results

### 3.1. Copy Number Variation of Hif-1α in Fish Lineages

**Figure 1:**
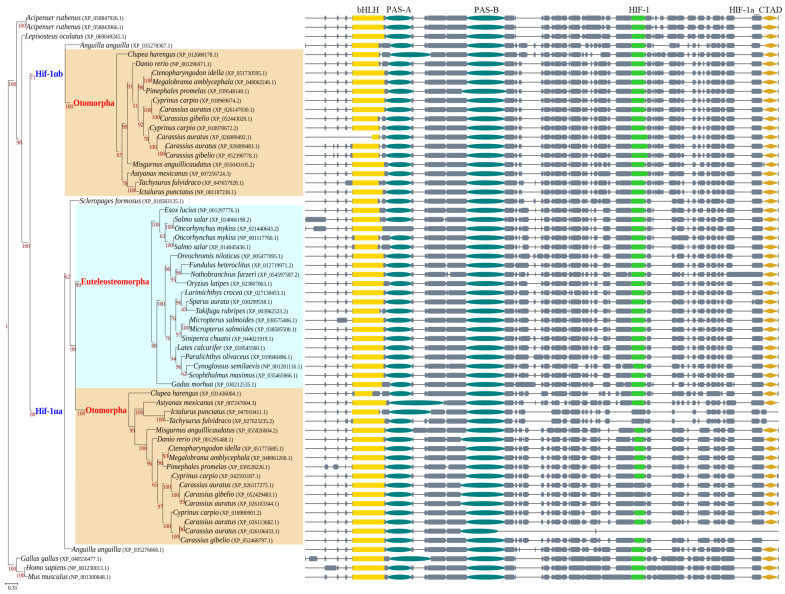
Phylogenetic analysis and domain architecture of HIF-1α in fishes. The maximum likelihood tree (**left**) depicts the evolutionary relationships of HIF-1α sequences from 33 fish species, mouse, chicken, and human. Otomorphs generally retain Hif-1αa and Hif-1αb resulting from TGD, whereas most euteleosts possess only a single Hif-1αa copy. Schematics (**right**) show the domain organization of corresponding sequences. The colored blocks represent five conserved domains predicted by CD-search: basic helix-loop-helix (bHLH), Per-Arnt-Sim (PAS, including PAS-A and PAS-B), hypoxia-inducible factor-1 (HIF-1), and HIF-1 alpha C terminal transactivation domain (HIF-1a_CTAD). The ODD domain, as mentioned in the text, is typically lengthy and fully encompasses the HIF-1 domain displayed here. Gray blocks represent aligned regions, and horizontal lines indicate gaps in the sequence alignment.

We performed BLASTP searches using the human HIF-1α protein sequence as a query to identify homologous sequences across 33 fish species and reconstructed a phylogenetic tree. Based on the tree topology and syntenic relationships, we determined the distribution of HIF-1α orthologs. As shown in Figure 1, otomorphs generally possess two Hif-1α copies (Hif-1αa and Hif-1αb), which likely originated from the TGD event. In contrast, euteleosts possess only a single Hif-1αa copy, indicating that Hif-1αb was lost in their common ancestor. The more recent common ancestors of goldfish and common carp, as well as of rainbow trout and Atlantic salmon, experienced additional WGD events, resulting in expanded Hif-1α copy numbers in these species. Goldfish possess more Hif-1α copies than common carp, suggesting that subsequent small-scale duplication events occurred in goldfish.

The vast majority of the identified sequences contained a complete set of five characteristic domains, suggesting they are functional. These functions include forming stable heterodimers with ARNT under hypoxia, binding to HREs, recruiting transcriptional coactivators EP300/CREBBP, and initiating the transcription of downstream genes. Notably, five Hif-1αa sequences from otomorphs lacked the canonical HIF-1 domain (cdd accession: pfam11413). The corresponding regions were not alignment gaps, indicating genuine sequence divergence. Because the C-terminal LXXLAP motif, which is critical for HIF-1α stability, resides within this domain, we speculate that its absence or alteration in these sequences could increase Hif-1αa protein stability under normoxic conditions.

### 3.2. Mutations in the LXXLAP Motifs

**Figure 2:**
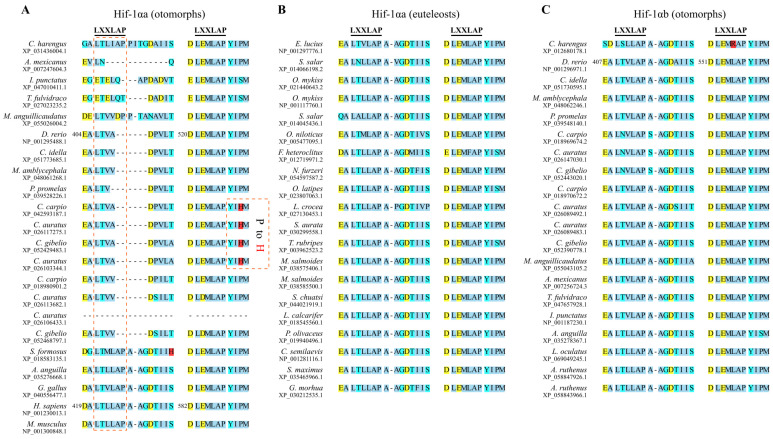
Partial multiple sequence alignment of HIF-1α sequences surrounding the two LXXLAP motifs. (**A**) Hif-1αa sequences of otomorphs and HIF-1α sequences of chicken, human, and mouse; (**B**) Hif-1αa sequences of euteleosts; (**C**) Hif-1αb sequences of otomorphs. Background colors denote amino acid properties: nonpolar (sky blue), polar neutral (cyan), basic (red), acidic (yellow). For sequences of the zebrafish and human, numbers on the left side indicate the start positions of respective regions. Key mutations in the Hif-1αa proteins are indicated by rectangular dashed boxes.

Because PHD2 recognizes and hydroxylates proline residues in HIF-1α by binding to the LXXLAP motifs, we screened our multiple sequence alignment for variations in these motifs. We found that the N-terminal LXXLAP motif was variable and poorly conserved in otomorphs Hif-1αa sequences, whereas the C-terminal motif remained highly conserved (Figure 2A). In contrast, both LXXLAP motifs were highly conserved in euteleost Hif-1αa sequences and in otomorphs Hif-1αb sequences (Figure 2B,C). Furthermore, in four Hif-1αa sequences from goldfish, common carp, and gibel carp (*Carassius gibelio*, originated from gynogenesis of goldfish), we identified a proline-to-histidine (P-to-H) substitution adjacent to the conserved proline in the C-terminal of the ODD domain (Pro_CODD_) (Figure 2A). This substitution alters the local charge and is predicted to impair PHD2 binding. Collectively, these findings suggest that specific Hif-1α paralogs in otomorphs, particularly in goldfish and its closely related species, have evolved increased stability under normoxia.

### 3.3. Mutations in the LXXLAP Motif Impair Phd2 Binding Affinity

**Figure 3:**
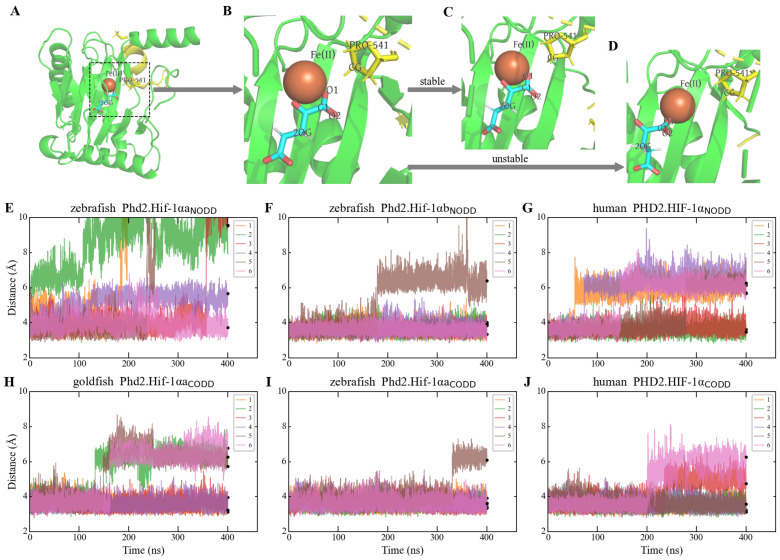
Comparison of binding stability of different complexes using MD simulations. The structural diagrams (**A**–**D**) illustrate the differences between stable and unstable replicas during MD simulations. The complexes displayed here are all goldfish Phd2.Hif-1αa_CODD_. Phd2 is colored green and the CODD peptide yellow. (**A**,**B**) are taken from the first frame, while (**C**,**D**) are from the final frame of the 400 ns simulation. (**E**–**J**) Time-dependent changes in the CG-O1 distance for the six independent simulation replicas of each complex. This distance serves as a metric for binding stability. Instability is defined as a sustained increase in CG-O1 distance.

By analyzing the PHD2-HIF-1α crystal structure (PDB: 5L9B [40]), we found that the CG atom (target atom of hydroxylation) of Pro_CODD_ is in close proximity to the O1 atom of 2-oxoglutarate (2OG) (Figure 3A,B; Supplementary Figure S1). We therefore used the distance between these atoms (CG-O1) as a metric for binding strength in MD simulations (Figure 3C,D). We modeled the structures of several complexes: zebrafish Phd2.Hif-1αa_CODD_, Phd2.Hif-1αb_NODD_, and a mutated Phd2.Hif-1αa_NODD_; goldfish Phd2.Hif-1αa_CODD_; and human PHD2.HIF-1α_NODD_ and PHD2.HIF-1α_CODD_. We conducted MD simulations for each complex, with six replicas per complex. Among the three NODD complexes, zebrafish Phd2.Hif-1αa_NODD_ complex was most unstable: in five out of six replicas, Pro_NODD_ and 2OG dissociated (Figure 3E). This confirms that Phd2 cannot bind effectively to the non-conserved LXXLAP motif in Hif-1αa_NODD_ (Figure 2A).

Among the CODD complexes, goldfish Phd2.Hif-1αa_CODD_ was the least stable (Figure 3H), although the effect was less pronounced than for the zebrafish NODD complex. In all three unstable replicas of the goldfish complex, the separation between Pro_CODD_ and 2OG occurred earlier (before 200 ns) than in unstable replicas of the zebrafish and human CODD complexes (Figure 3I,J), indicating substantially weaker stability. The loop regions of PHD2 that interact with the NODD/CODD peptides were highly conserved across the three species examined (Supplementary Figures S2 and S3) [41]. Therefore, the instability of goldfish Phd2.Hif-1αa_CODD_ complex is directly attributable to the P-to-H substitution near Pro_CODD_ (Figure 2A).

We also found that the human PHD2.HIF-1α_NODD_ was less stable than PHD2.HIF-1α_CODD_ (Figure 3G,J), consistent with previous experimental evidence that the NODD plays a secondary role to the CODD in regulating oxygen-dependent HIF-α degradation [42]. This consistency with established literature supports the validity of our MD simulation approach and the use of the CG-O1 distance as a reliable metric for characterizing PHD2-LXXLAP binding affinity.

### 3.4. Functional Divergence of Hif-1αa and Hif-1αb

**Figure 4:**
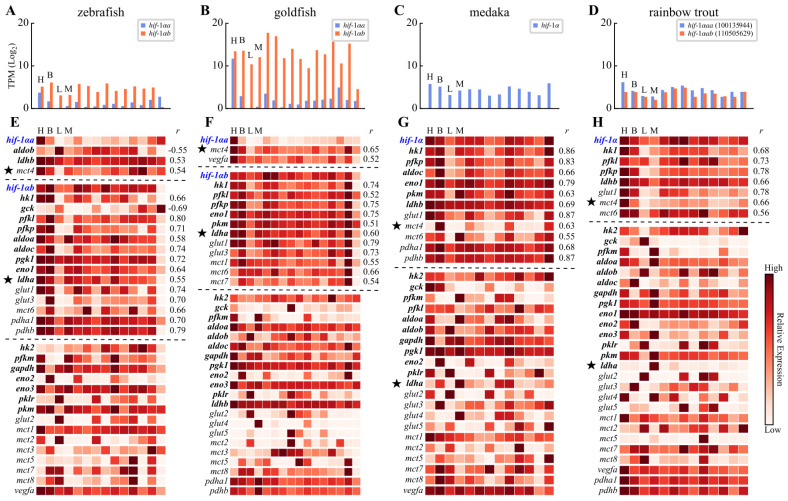
Tissue expression profiles of *hif-1α* genes and putative HIF target genes in four fish species. Letters H, B, L, M represent heart, brain, liver and muscle, respectively. The expression levels of *hif-1α* paralogs in different tissues of four fish species are presented in panels (**A**–**D**). The Pearson correlation coefficient (*r*) between the expression profile of a downstream gene and a *hif-1α* gene is shown if the correlation is statistically significant (*p* < 0.05, listed in panels (**E**–**H**)). Genes encoding key glycolytic enzymes are highlighted in bold. The positions of the *ldha* and *mct4* genes in the heatmaps are prominently marked with pentagram symbols (“★”). TPM, transcripts per million.

To investigate the functional consequences of the LXXLAP motif variations, we identified orthologs of known HIF-1α target genes in fishes and analyzed RNA-seq data from zebrafish, goldfish, medaka, and rainbow trout (using two BioProjects per species; see Supplementary Table S3 for target gene details). We assessed the similarity of gene expression profiles using Pearson correlation coefficients. The expression profiles of *hif-1αa* and *hif-1αb* were significantly divergent in zebrafish and goldfish. Specifically, *hif-1αa* expression was highest in the heart and low in other tissues, whereas *hif-1αb* was ubiquitously expressed (Figure 4A,B). Although rainbow trout possesses two *hif-1α* copies, they originated from a recent duplication of *hif-1αa* (Figure 1) and consequently exhibited highly correlated expression profiles (Figure 4D; *r* = 0.74, *p* < 0.01).

Owing to its tissue-specific expression, only a small subset of downstream genes exhibited expression profiles significantly correlated with *hif-1αa* in zebrafish or goldfish (Figure 4E,F). This included few genes encoding key glycolytic enzymes, suggesting that Hif-1αa may not be the primary regulator of basal glycolytic gene expression. Notably, however, the *ldha* genes in both zebrafish and goldfish were highly expressed in the heart—at levels exceeding those in muscle. This pattern is atypical, as lactate dehydrogenase A (LDHA) is predominantly a muscle-specific enzyme, as seen in medaka and rainbow trout (Figure 4G,H). Although the correlation between *ldha* and *hif-1αa* was not statistically significant, the high cardiac expression of *ldha* suggests its potential regulation by Hif-1αa. Conversely, the expression profile of *mct4* was significantly correlated with that of *hif-1αa* in both species. Monocarboxylate transporter 4 (MCT4) is a low-affinity, high-capacity lactate transporter. This co-expression with *hif-1αa* suggests a mechanism whereby Hif-1αa regulates lactate efflux to prevent toxic intracellular accumulation during glycolysis.

## 4. Discussion

**Figure 5:**
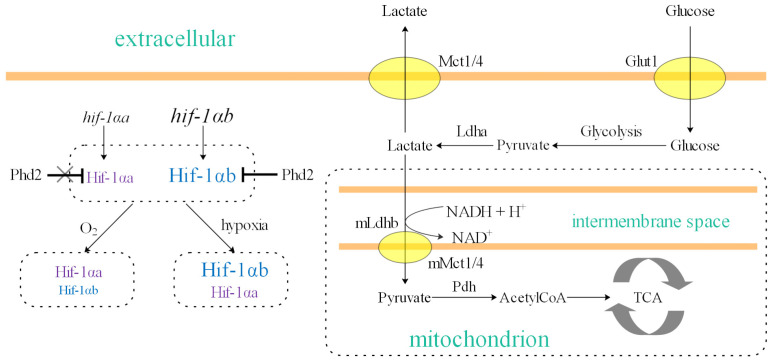
Proposed model for Hif-1αa-mediated enhancement of hypoxia tolerance in otomorphs. Under normoxia, the mutated Hif-1αa protein is stable and drives the constitutive expression of its target genes, including *ldha* and *mct4*. This pre-adapts the cell by maintaining high levels of glycolytic enzyme (Ldha) and lactate transporter (Mct4). Upon exposure to hypoxia, the cell can immediately sustain high-rate anaerobic glycolysis. Lactate produced from glycolysis is efficiently exported by Mct4, preventing intracellular acidification and toxicity, thereby enhancing hypoxic survival. The relative expression levels of *hif-1αa* and *hif-1αb* genes, as well as the relative concentrations of the corresponding proteins under normoxia and hypoxia (indicated by font size), were inferred based on the findings of this study. The fundamental glycolytic and lactate shuttle pathways were adapted from [46].

Under hypoxic stress, cells suppress oxidative phosphorylation to reduce oxygen consumption and consequently rely more heavily on glycolysis for energy production. As glycolysis yields less ATP per glucose molecule than oxidative phosphorylation, cells must increase their glucose uptake, often by upregulating glucose transporters [5,43,44]. The net reaction from glucose to pyruvate consumes cytosolic NAD^+^. To maintain glycolytic flux, pyruvate is reduced to lactate by lactate dehydrogenase (LDH), which simultaneously regenerates NAD^+^ from NADH [45,46]. LDH is a homo- or hetero-tetramer composed of LDHA and LDHB subunits (encoded by LDHA and LDHB genes, respectively), ultimately form five different isozymes: LDH1 (B4), LDH2 (AB3), LDH3 (A2B2), LDH4 (A3B) and LDH5 (A4) [47,48]. Typically, the LDHA subunit (muscle-type) catalyzes the reduction of pyruvate to lactate and is predominantly expressed in skeletal muscle. In contrast, the LDHB subunit (heart-type) catalyzes the oxidation of lactate to pyruvate and is the primary form in the heart [45]. In this study, we observed that *ldha* expression in the hearts of zebrafish and goldfish was higher than in their muscle, a pattern opposite to the typical tissue distribution. We propose that this atypical, high cardiac *ldha* expression is induced by the normoxically stable Hif-1αa (Figure 5). This adaptation suggests that otomorphs have evolved a constitutively enhanced capacity for anaerobic glycolysis in the heart.

Intense anaerobic glycolysis, however, leads to lactate accumulation. To mitigate lactate toxicity, cells utilize MCT4 for high-capacity lactate efflux [46,49]. We found that *mct4* expression was highest in the hearts of zebrafish and goldfish, correlating with elevated *ldha* levels. Furthermore, *mct4* was highly expressed in other tissues like the brain and muscle. This co-expression pattern implies that lactate produced in these tissues can be efficiently exported into the bloodstream (Figure 5), facilitating an inter-tissue lactate shuttle. This pre-adapted metabolic framework is likely a key factor enhancing hypoxia tolerance in otomorphs [5]. In contrast, *mct4* expression in medaka and rainbow trout is more tissue-specific and is not inducible by hypoxia or exercise in tissues like liver and muscle [50]. The consequently less efficient lactate shuttle in these euteleosts may limit their glycolytic capacity and contribute to their poorer hypoxia tolerance.

While enhanced glycolytic potential and lactate shuttling are beneficial for coping with hypoxic challenges, their excessive activation under normoxic conditions can be detrimental, leading to both nutrient waste and an increased susceptibility to severe pathological conditions [51]. Otomorphs appear to elegantly resolve this trade-off through differential gene expression. Specifically, their *hif-1αa* gene is highly expressed only in the heart, and even this elevated expression level is considerably lower than that of *hif-1αb* (note that the expression levels in Figure 4A–D are log-transformed; thus, small differences on the y-axis correspond to large differences in actual TPM values). Consequently, although Hif-1αa protein exhibits markedly increased stability under normoxia, its steady-state concentration remains limited (Figure 5). This prevents an excessively high basal glycolytic rate, while allowing rapid metabolic reprogramming when hypoxia occurs. Under sustained hypoxia, further elevation of glycolysis can be achieved through the stabilized Hif-1αb protein, reaching an even higher level of glycolytic activity.

## 5. Conclusions

In this study, we demonstrated that the two Hif-1α paralogs resulting from TGD have been retained in otomorphs with significant functional divergence. Specifically, the Hif-1αa paralog has evolved increased stability under normoxia due to mutations disrupting its N-terminal LXXLAP motif. This stable Hif-1αa promotes the expression of key metabolic genes, including *ldha* and *mct4*, pre-adapting tissues like the heart, brain, and muscle for intense glycolysis. Coupled with a more efficient inter-tissue lactate shuttle compared to euteleosts, these adaptations enhance the glycolytic capacity of otomorphs, ensuring a continuous energy supply during hypoxic stress.

## Data Availability

The original contributions presented in the study are included in the article/Supplementary Materials, further inquiries can be directed to the corresponding authors.

## Supplementary Materials

The following supporting information can be downloaded at: https://www.mdpi.com/article/10.3390/ani16010119/s1, Table S1: Protein components of complexes used for MD simulations. Table S2: BioProjects of four species used for gene expression analyses. Figure S1: Distances between CG atom of ProCODD and atoms of 2OG and Mn(II) (PDB ID: 5L9B). Figure S2: Loop region in PHD2 (colored magenta) that directly interacts with NODD/CODD (colored yellow). Figure S3: Partial multiple sequence alignment of PHD2 sequences corresponding to the loop region that directly interacts with NODD/CODD. Table S3: Detailed information of downstream genes in four fish species.
