# Supplementary material for: Mutated Hif-1αa Proteins with Increased Stability Under Normoxic Conditions Enhance Hypoxia Tolerance of Otomorphs by Promoting Glycolysis and Lactate Shuttle
Source: Animals (Basel). 2025 Dec 31;16(1):119. doi: 10.3390/ani16010119 (PMC12784922; doi:10.3390/ani16010119)
Supplement: Supplementary file 1 [file animals-16-00119-s001.zip › supplementary material v0.1.pdf]

Supplementary Material

Mutated Hif-1αa Proteins with Increased Stability Under Normoxic Conditions Enhance Hypoxia Tolerance of Otomorphs by Promoting Glycolysis and Lactate Shuttle

Xianzong Wang [1,2,*], Junli Yan [3], Huili Zhai [1], Jiali Guo [1], Xueyi Wang [1], Qing Liu [1,2] and Shaozhen Liu [1,2,*]

[1] College of Animal Science, Shanxi Agricultural University, Taigu District, Jinzhong 030801, China; zhaihuili_l@163.com (H.Z.); 17302250363@163.com (J.G.); 15685488103@163.com (X.W.); liuqing_sxau@126.com (Q.L.)
[2] Shanxi Key Laboratory of Animal Genetics Resource Utilization and Breeding, Shanxi Agricultural University, Taigu District, Jinzhong 030801, China
[3] College of Urban and Rural Construction, Shanxi Agricultural University, Taigu District, Jinzhong 030801, China; y_jl2020@163.com
* Correspondence: xianzong_wang@126.com (X.W.); shmily8316@126.com (S.L.)

Table S1. Protein components of complexes used for MD simulations.

Organism	Protein (accession No.)	Region for structure prediction (aa)	Region for MD simulation (aa)	Complex name[a]
zebrafish	Hif-1αa (NP_001295488.1)	389-429	404-421<br>EA**LTVADP**VLTLDINITD	Phd2.Hif-1αa$_{NODD}$
	Phd2 (XP_002664281.2)	full length	91-306	
zebrafish	Hif-1αa (NP_001295488.1)	506-546	519-536<br>LD**LEMLAP**YIPMDDDFQL	Phd2.Hif-1αa$_{CODD}$
	Phd2 (XP_002664281.2)	full length	91-306	
zebrafish	Hif-1αb (NP_001296971.1)	394-434	407-424<br>EA**LTVLAP**AAGDAIISLD	Phd2.Hif-1αb$_{NODD}$
	Phd2 (XP_002664281.2)	full length	91-306	
goldfish	Hif-1αa (XP_026103344.1)	521-561	534-551<br>LD**LEMLAP**YIHMEDDYQL	Phd2.Hif-1αa$_{CODD}$
	Phd2 (XP_026133603.1)	full length	94-320	
human	HIF-1α (NP_001230013.1)	406-446	419-436<br>DA**LTLLAP**AAGDTIISLD	PHD2.HIF-1α$_{NODD}$
	PHD2 (NP_071334.1)	full length	188-409	
human	HIF-1α (NP_001230013.1)	568-608	581-598<br>LD**LEMLAP**YIPMDDDFQL	PHD2.HIF-1αa$_{CODD}$
	PHD2 (NP_071334.1)	full length	188-409	

[a] Although we did not include the names of 2OG and Fe(II), they were present in each final complex for MD simulations.

Table S2. BioProjects of four species used for gene expression analyses.

Organism	Bioproject No.	Average bases per run/G	Library layout	Sampled tissues
zebrafish	PRJEB37848	4.5	paired	brain, eye, gill, gut, heart, liver, muscle, skin
	PRJNA255848	10.3	paired	bones, brain, embryo, gills, heart, intestine, kidney, liver, muscle, ovary, testis, unfertilized_eggs
goldfish	PRJNA580146	7.2	paired	brain, eye, gill, head, heart, intestine, kidney, larvae, liver, muscle, pectoral_fin, skin, spleen, testis
	PRJNA833750	14.6	paired	brain, heart, hypothalamus-pituitary, kidey, liver, muscle, overy, spleen, testis
medaka	PRJEB37848	4.5	paired	brain, eye, gill, gut, heart, liver, muscle, skin
	PRJNA255889	6.2	paired	bones, brain, embryo, gills, heart, intestine, kidney, liver, muscle, ovary, testis
rainbow trout	PRJEB37848	4.5	paired	brain, eye, gill, gut, head_kidney, heart, kidney, liver, muscle, pyloric_caeca, skin, spleen
	PRJEB57191	9.7	paired	brain, fast_muscle_tissue, gill_filament, gonad_male, head_kidney, liver, terminal_part_of_digestive_tract

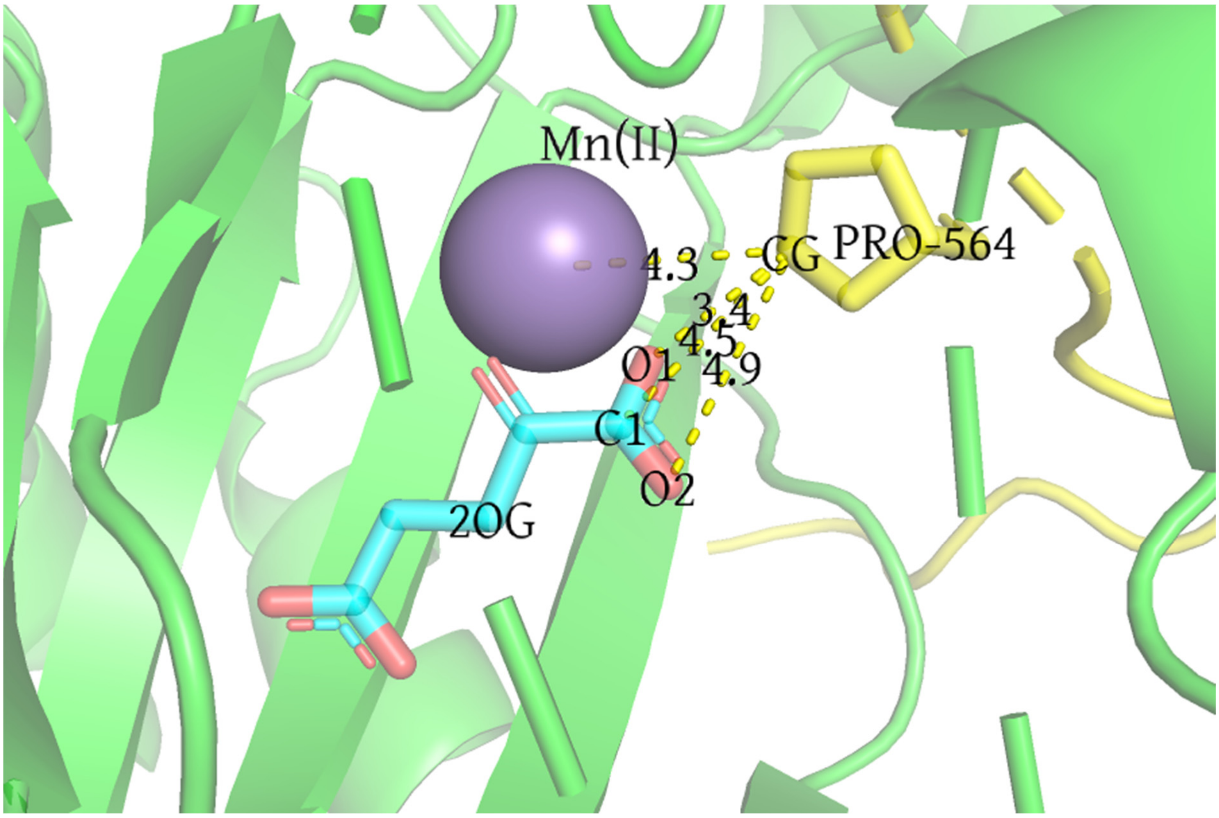

Figure S1. Distances between CG atom of Pro$_{CODD}$ and atoms of 2OG and Mn(II) (PDB ID: 5L9B).

Distance measurements were made with PyMOL and in angstroms (Å).

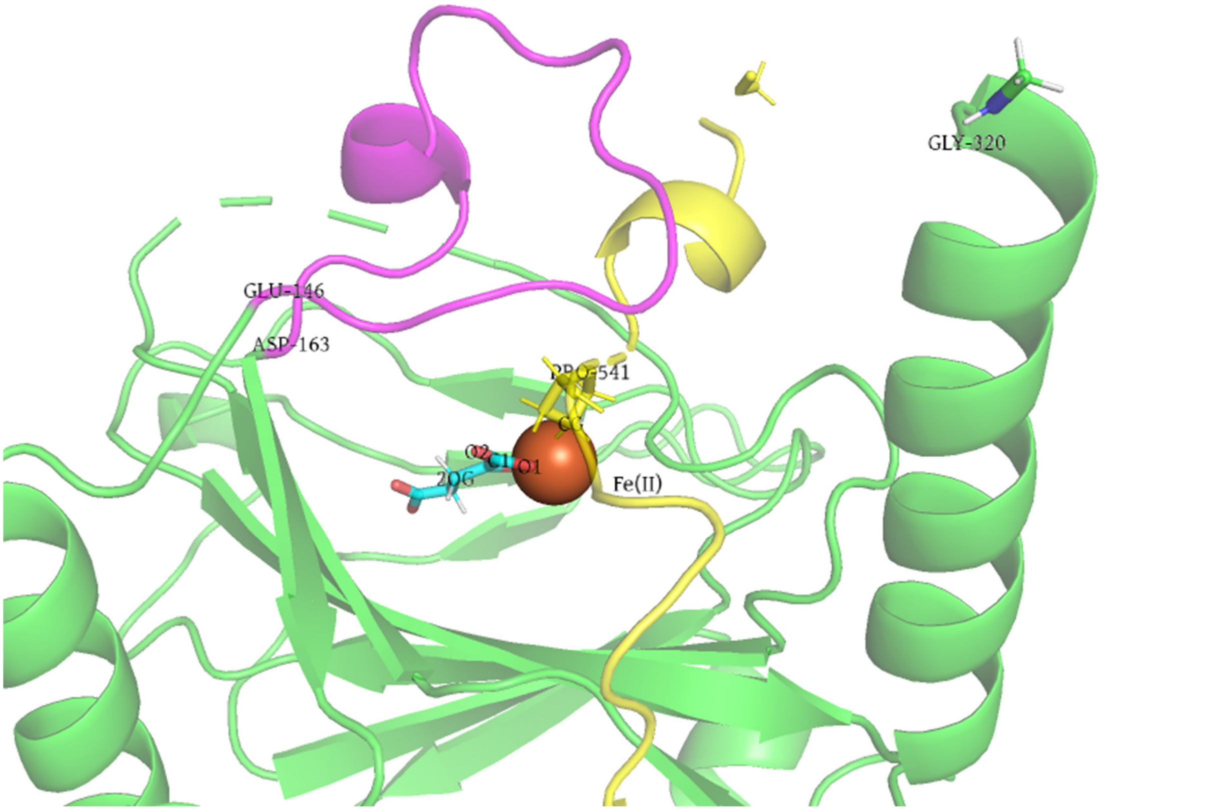

Figure S2. Loop region in PHD2 (colored magenta) that directly interacts with NODD/CODD (colored yellow).

The complex displayed here is the goldfish Phd2.Hif-1αa_{CODD}.

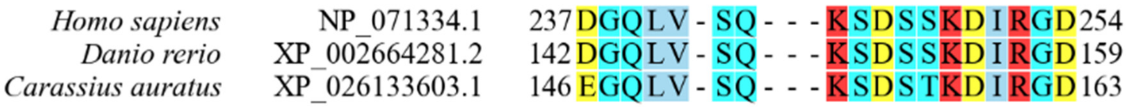

Figure S3. Partial multiple sequence alignment of PHD2 sequences corresponding to the loop region that directly interacts with NODD/CODD.
